# Supplementary material for: Amyloid β oligomers suppress excitatory transmitter release via presynaptic depletion of phosphatidylinositol-4,5-bisphosphate
Source: Nat Commun. 2019 Mar 13;10:1193. doi: 10.1038/s41467-019-09114-z (PMC6416269; doi:10.1038/s41467-019-09114-z)
Supplement: Supplementary file 1 — Supplementary Information [file 41467_2019_9114_MOESM1_ESM.pdf]

Supplementary Materials for

Amyloid β oligomers suppress excitatory transmitter release via presynaptic depletion of phosphatidylinositol-4,5-bisphosphate

Yang He, Mengdi Wei, Yan Wu, Huaping Qin, Weinan Li, Xiaolin Ma, Jingjing Cheng, Jinshuai Ren, Ye Shen, Zhong Chen, Binggui Sun, Fu-De Huang, Yi Shen, and Yu-Dong Zhou

*Correspondence: yudongzhou@zju.edu.cn (Y.-D.Z.), yshen2@zju.edu.cn (Y.S.), or Fude Huang: huangfd@sari.ac.cn (F.-D.H.)

This PDF file includes:

Figs. S1 to S15

Materials and Methods

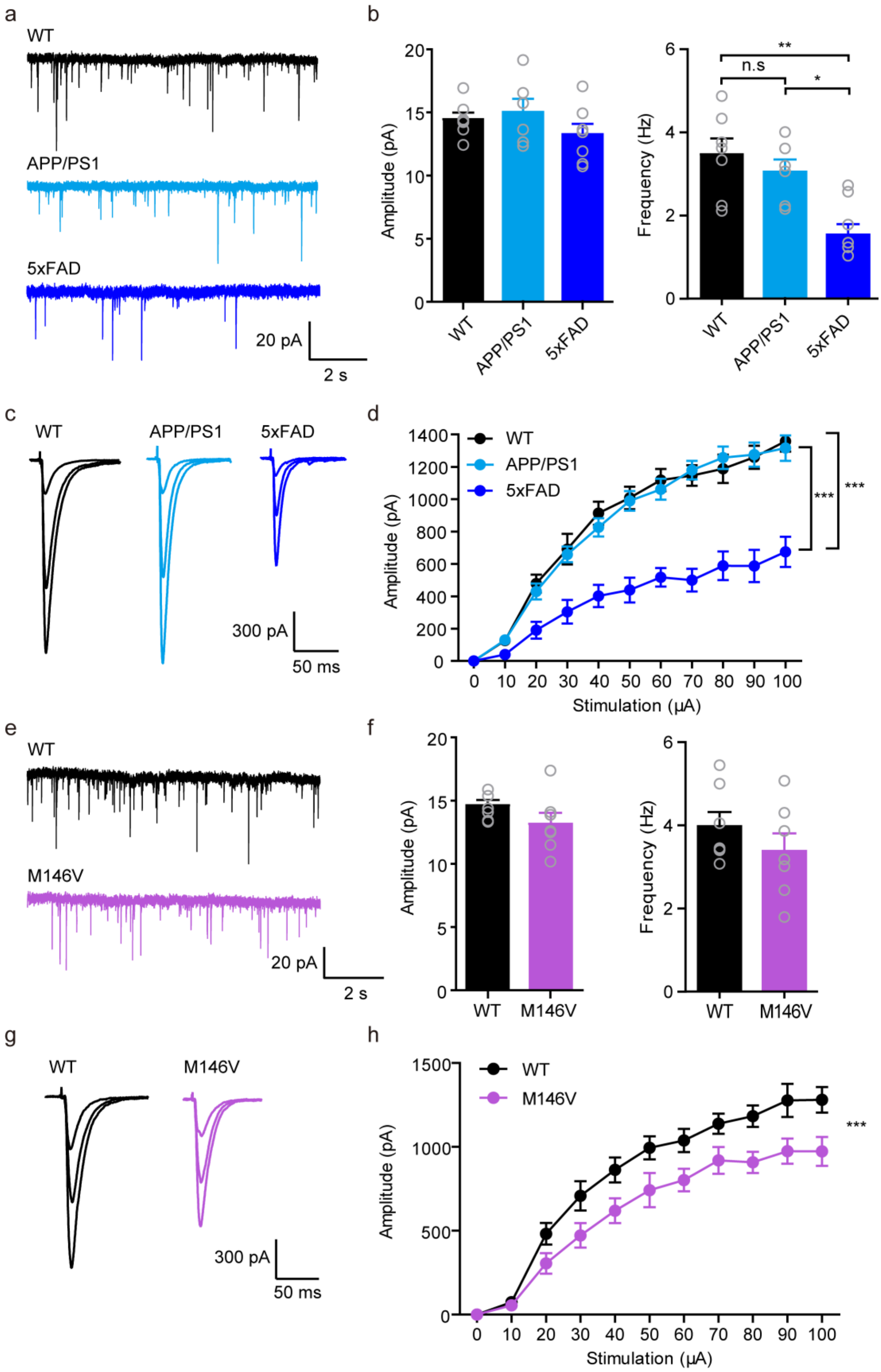

Supplementary Figure 1 Excitatory synaptic transmission deficits in AD mouse models. **a, b** Representative traces (**a**) of mEPSCs in CA1 pyramidal neurons and

quantification (**b**) of mEPSC amplitude (left) and frequency (right) in 4-month-old WT, APP/PS1, and 5xFAD mice. One-way ANOVA with post hoc Dunnett's test; $F_{(2,17)}$ = 1.19 (amplitude); $F_{(2,17)}$ = 9.484 (frequency); *, P < 0.05; **, P < 0.01; N = 6-7 per group. **c,d** Representative traces of SC-CA1 EPSCs evoked by stimulus intensities of 10, 30, and 100 μA (**c**) and quantification of EPSC amplitude to stimulus intensity (**d**) in 4-month-old WT, APP/PS1, and 5xFAD mice. Two-way ANOVA with post hoc Bonferroni test; animal, $F_{(2,198)}$ = 168.1, P < 0.001; stimulation, $F_{(10,198)}$ = 107.6, P < 0.001; ***, P < 0.001; N = 6-8 per group. **e, f** Representative traces (**e**) of mEPSCs in CA1 pyramidal neurons and quantification (**f**) of mEPSC amplitude (left) and frequency (right) in 6-month-old WT and M146V mice. *t* test; P > 0.05; N = 7 per group. **g, h** Representative traces of SC-CA1 EPSCs evoked by stimulus intensities of 20, 40, and 100 μA (**g**) and quantification of EPSC amplitude to stimulus intensity (**h**) in 6-month-old WT and M146V mice. Two-way ANOVA with post hoc Bonferroni test; animal, $F_{(1,143)}$ = 49.34, P < 0.001; stimulation, $F_{(10,143)}$ = 70.41, P < 0.001; ***, P < 0.001; N = 7-8 per group. Data are mean ± SEM. Source data are provided as a Source Data file.

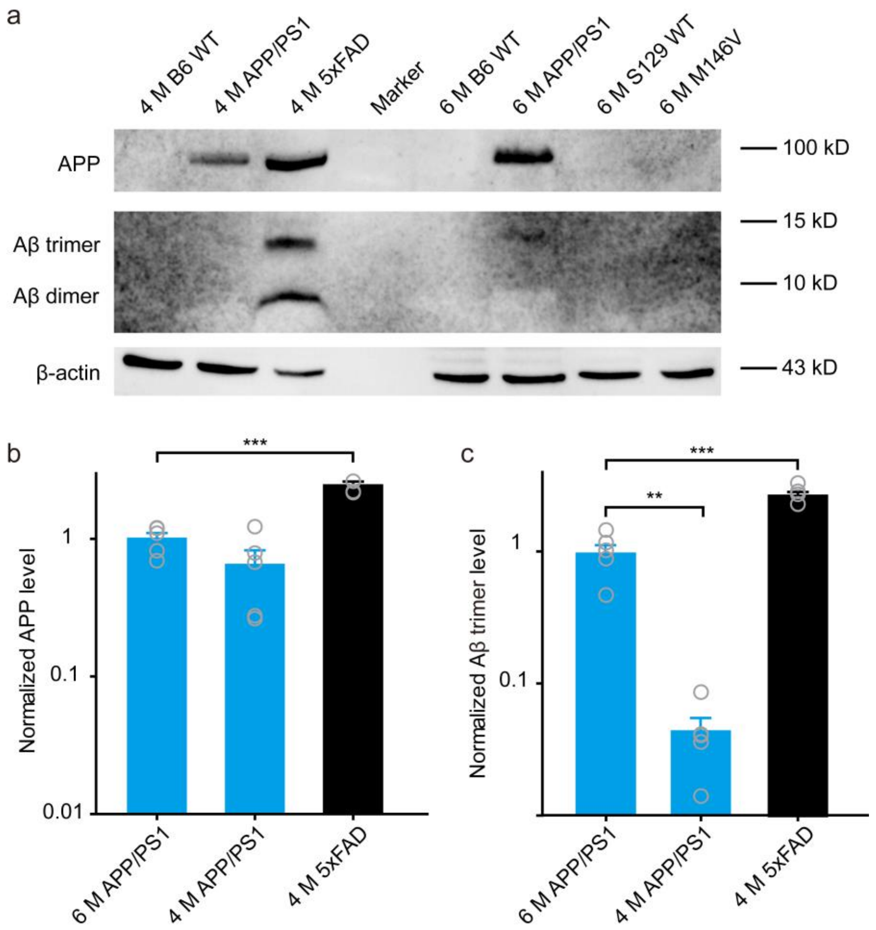

Supplementary Figure 2 Oligomeric Aβ levels are greatly enhanced in 4-month-old 5xFAD and 6-month-old APP/PS1 mice in comparison to 4-month-old APP/PS1 mice. **a** Representative Western blots of APP and Aβ oligomers (trimer/dimer) in 4-month-old (4 M) WT, APP/PS1, and 5xFAD mice and 6-month-old (6 M) WT, APP/PS1, S129 WT, and M146V mice. **b, c** Quantification of APP (**b**) and Aβ trimer (**c**) in 4 M 5xFAD, 4 M APP/PS1, and 6 M APP/PS1 mice. One-way ANOVA with post hoc Dunnett's test; $F_{(2,12)} = 39.025$ in **b**; $F_{(2,12)} = 86.069$ in **c**; **, $P < 0.01$; ***, $P < 0.001$; $N = 5$ per group. Data are mean ± SEM. Source data are provided as a Source Data file.

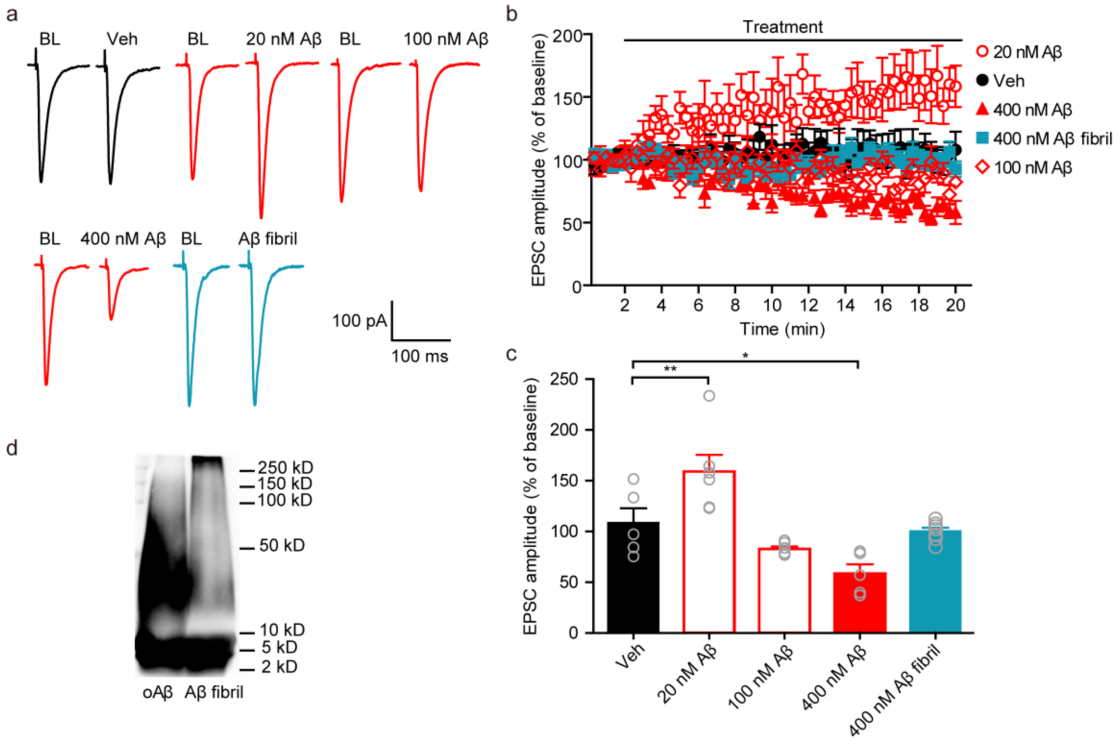

Supplementary Figure 3 Regulation of evoked responses at the SC-CA1 synapse by Aβ fibrils and oligomers at various concentrations. **a, b** Representative traces (**a**) and the time course (**b**) of evoked EPSCs at the SC-CA1 synapse before (baseline, BL) and after DMSO (vehicle, Veh), oligomeric $Aβ_{42}$ (20, 100, or 400 nM), or fibrillar $Aβ_{42}$ (400 nM) treatment. **c** Quantification of EPSC amplitudes recorded in the last 1 min in **b**. One-way ANOVA with post hoc Dunnett's test; $F_{(4,24)}$ = 12.64; *, P < 0.05; **, P < 0.01; N = 5-7 per group. **d** Representative Western blots of synthetic oligomeric $Aβ_{42}$ (oAβ) and fibrillar $Aβ_{42}$ (Aβ fibril). Data are mean ± SEM. Source data are provided as a Source Data file.

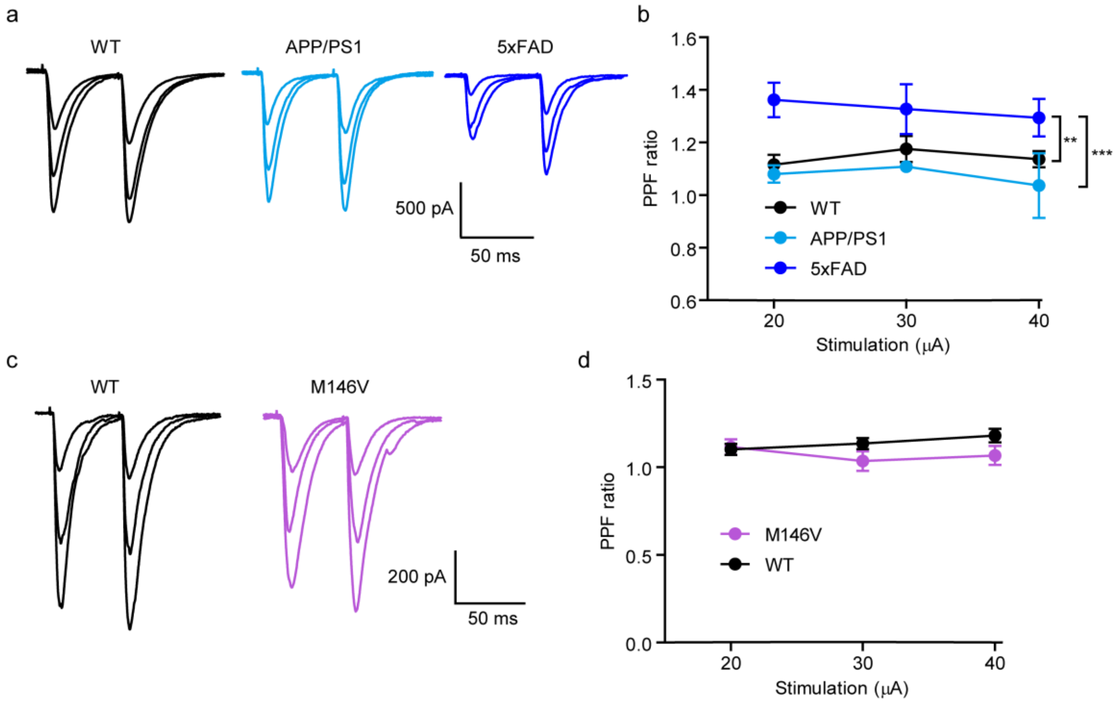

Supplementary Figure 4 PPF at the SC-CA1 synapse is strongly increased in 4-month-old 5xFAD mice, but not in 6-month-old M146V mice. **a, b** Representative traces (**a**) and quantification (**b**) of PPF in response to stimulus intensities of 20, 30, and 40 μA in 4-month-old WT, APP/PS1, and 5xFAD mice. Two-way ANOVA with post hoc Bonferroni test; animal, $F_{(2,51)} = 11.27$, $P < 0.001$; stimulation, $F_{(2,51)} = 0.3558$, $P < 0.001$; **, $P < 0.01$; ***, $P < 0.001$; N = 6-8 per group. **c, d** Representative traces (**c**) and quantification (**d**) of PPF in response to stimulus intensities of 20, 30, and 40 μA in 6-month-old WT and M146V mice. Two-way ANOVA with post hoc Bonferroni test; animal, $F_{(1,42)} = 3.745$, $P = 0.07$; stimulation, $F_{(2,42)} = 0.4371$, $P = 0.65$; $P > 0.05$; N = 7-9 per group. Data are mean ± SEM. Source data are provided as a Source Data file.

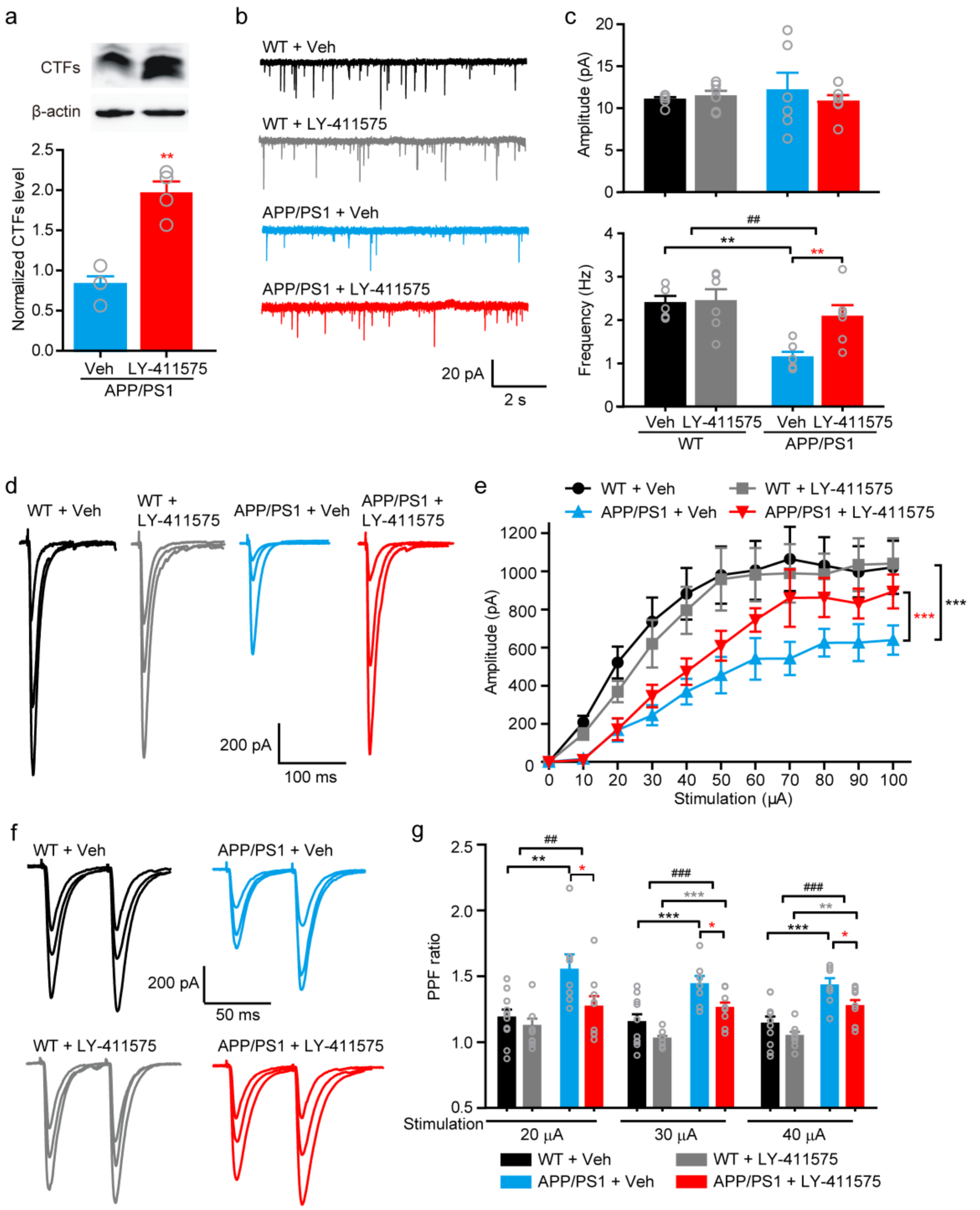

Supplementary Figure 5 Reducing Aβ production by γ-secretase inhibitor LY-411575 partially rescues excitatory synaptic deficits in 6-7-month-old APP/PS1 mice. **a** Representative Western blots (upper) and quantification (bottom) of hippocampal CTFs in APP/PS1 mice treated with vehicle (Veh) or LY-411575. *t* test; **, $P < 0.01$; $N = 4$ per group. **b, c** Representative traces (**b**) of mEPSCs in CA1 pyramidal neurons

and quantification (**c**) of mEPSC amplitude (upper) and frequency (bottom) in WT and APP/PS1 mice treated with vehicle (Veh) or LY-411575. Two-way ANOVA with post hoc Bonferroni test; upper panel in **c**: animal, $F_{(1,29)}$ = 0.0391, P = 0.845; treatment, $F_{(1,29)}$ = 0.143, P = 0.709; bottom panel in **c**: animal, $F_{(1,29)}$ = 12.814, P = 0.002; treatment, $F_{(1,29)}$ = 4.733, P = 0.040; **, P < 0.01; $^{\#\#}$, P < 0.01 (APP/PS1 vs. WT); N = 5-6 per group. **d, e** Representative traces of SC-CA1 EPSCs evoked by stimulus intensities of 20, 30, and 100 μA (**d**) and quantification of EPSC amplitude to stimulus intensity (**e**) in WT + Veh, WT + LY-411575, APP/PS1 + Veh, and APP/PS1 + LY-411575 groups. Two-way ANOVA with post hoc Bonferroni test; group, $F_{(3,209)}$ = 38.257, P < 0.001; stimulation, $F_{(10,209)}$ = 46.014, P < 0.001; ***, P < 0.001; N = 5-7 per group. **f, g** Representative traces (**f**) and quantification (**g**) of PPF of SC-CA1 EPSCs evoked by stimulus intensities of 20, 30, and 40 μA in WT and APP/PS1 mice treated with Veh, or LY-411575. Two-way ANOVA with post hoc Bonferroni test; 20 μA: animal, $F_{(1,30)}$ = 11.471, P = 0.002; treatment, $F_{(1,30)}$ = 5.540, P = 0.025; 30 μA: animal, $F_{(1,30)}$ = 26.206, P < 0.001; treatment, $F_{(1,30)}$ = 9.045, P = 0.005; 40 μA: animal, $F_{(1,30)}$ = 27.079, P < 0.001; treatment, $F_{(1,30)}$ = 6.278, P = 0.018; *, P < 0.05; **, P < 0.01; ***, P < 0.001; $^{\#\#}$, P < 0.01; $^{\#\#\#}$, P < 0.001 (APP/PS1 vs. WT); N = 7-10 per group. Data are mean ± SEM. Source data are provided as a Source Data file.

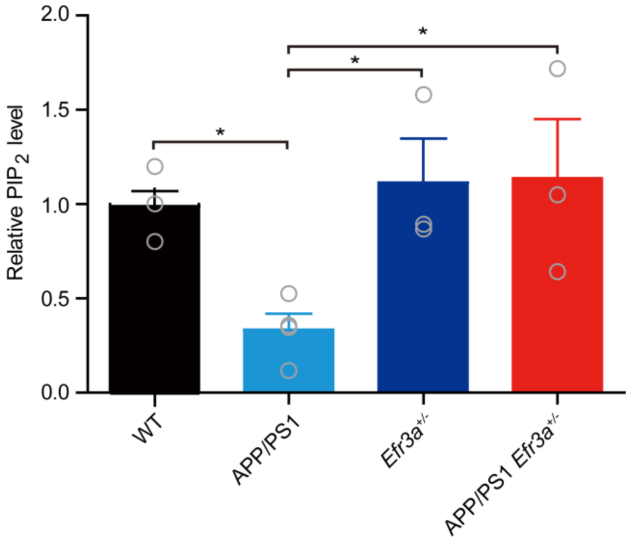

Supplementary Figure 6 Halving *Efr3a* copy number restores the decreased PIP_2 level measured with PIP_2 ELISA in APP/PS1 mice. One-way ANOVA with post hoc Dunnett's test; $F_{(3,10)} = 4.93$; *, $P < 0.05$; $N = 3$-4 per group. Data are mean ± SEM. Source data are provided as a Source Data file.

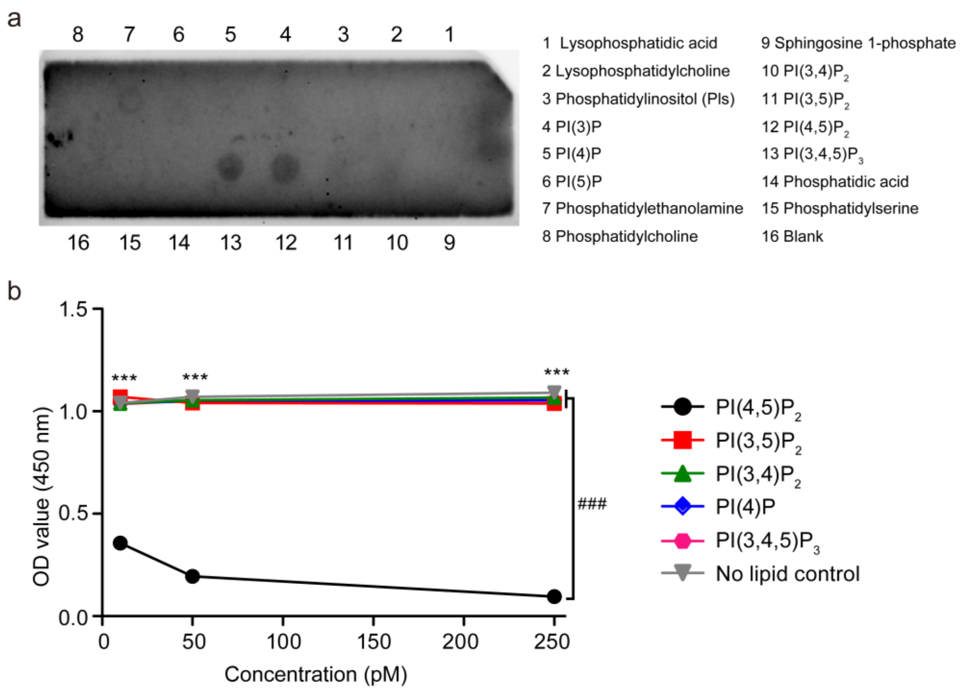

Supplementary Figure 7 Anti-PI(4,5)P_2 antibody and ELISA kit specificities. **a** Representative PIP strip showing the mouse anti-PI(4,5)P_2 antibody specifically recognizes PI(4,5)P_2 and PI(3,4,5)P_3. **b** Plot of colorimetric signals for various phosphatidylinositol phosphates at concentrations of 10, 50, and 250 pM. Two-way ANOVA with post hoc Bonferroni test; PIP species, $F_{(5,45)} = 4634.6$, $P < 0.001$; dose, $F_{(2,45)} = 12.527$, $P < 0.001$; ***, $P < 0.001$; $^{\#\#\#}$, $P < 0.001$; $N = 3\text{-}6$ per group. Data are mean ± SEM. Source data are provided as a Source Data file.

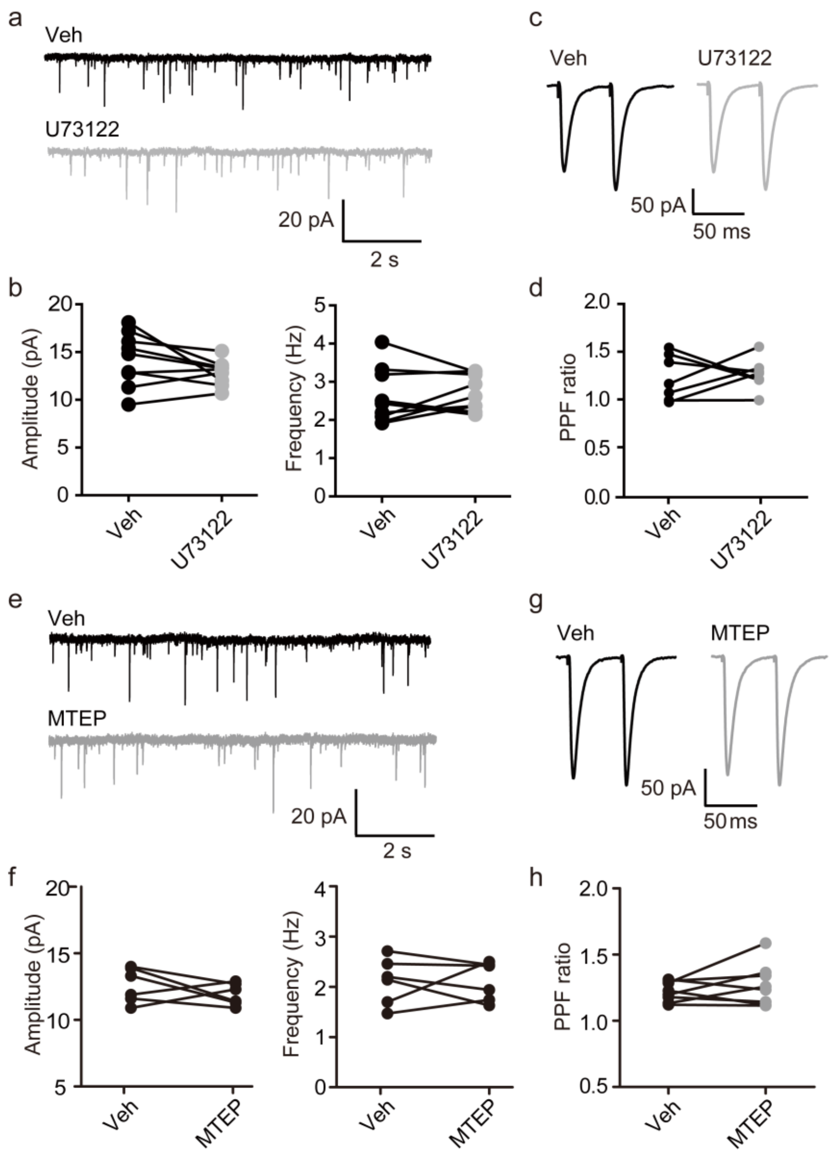

Supplementary Figure 8 Inhibiting PLC or mGluR5 has no influence on mEPSCs in CA1 pyramidal neurons and PPF at the SC-CA1 synapse in WT animals. **a, b** Representative traces (**a**) of mEPSCs in CA1 pyramidal neurons and quantification (**b**) of mEPSC amplitude (left) and frequency (right) in WT hippocampal slices before (Veh) and after U73122 treatment. *t* test; $P > 0.05$; $N = 9$ per group. **c, d** Representative traces (**c**) and quantification (**d**) of PPF of SC-CA1 EPSCs in WT mice before (Veh) and after U73122 treatment. *t* test; $P > 0.05$; $N = 7$ per group. **e, f** Representative

traces (**e**) of mEPSCs in CA1 pyramidal neurons and quantification (**f**) of mEPSC amplitude (left) and frequency (right) in WT hippocampal slices before (Veh) and after MTEP treatment. *t* test; $P > 0.05$; $N = 6$ per group. **g, h** Representative traces (**g**) and quantification (**h**) of PPF of SC-CA1 EPSCs in WT mice before (Veh) and after MTEP treatment. *t* test; $P > 0.05$; $N = 8$ per group. Data are mean ± SEM. Source data are provided as a Source Data file.

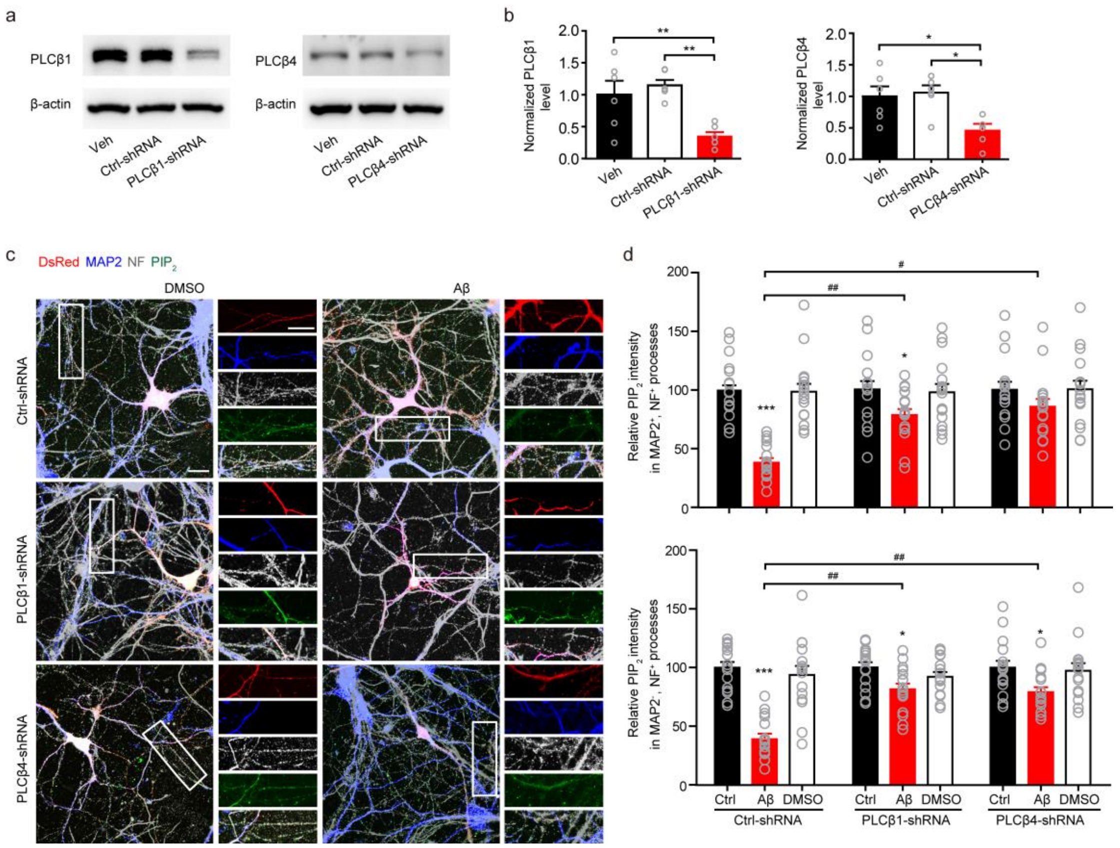

Supplementary Figure 9 Knocking down PLCβ1 or PLCβ4 prevents oligomeric Aβ-induced PIP_2 reduction in cultured hippocampal neurons. **a, b** Representative Western blots (**a**) and quantification (**b**) of PLCβ1 (left) and PLCβ4 (right) in primary hippocampal neurons treated with vehicle (Veh) or transfected with lentivirus carrying control (Ctrl)-shRNA, PLCβ1-shRNA, or PLCβ4-shRNA. One-way ANOVA with post hoc Dunnett's test; $F_{(2,15)} = 9.190$ (**b**, left); $F_{(2,14)} = 5.886$ (**b**, right); *, $P < 0.05$; **, $P < 0.01$; N = 5-6 per group. **c** Confocal images of primary hippocampal neurons infected with lentivirus carrying Ctrl-shRNA, PLCβ1-shRNA, or PLCβ4-shRNA showing the effect of DMSO or Aβ treatment (blank Ctrl treatment not shown) on colocalization of PIP_2, MAP2, and neurofilament (NF) along neuronal processes. Bars, 20 μm. **d** Quantification of relative PIP_2 intensity in dendrites (upper panel) and axons (bottom

panel) of lentiviral-infected, DsRed positive neurons. Two-way ANOVA with post hoc Bonferroni test; upper panel: cell type, $F_{(2,144)} = 5.693$, $P = 0.004$; treatment, $F_{(2,144)} = 25.666$, $P < 0.001$; bottom panel: cell type, $F_{(2,135)} = 6.999$, $P < 0.001$; treatment, $F_{(2,135)} = 33.85$, $P < 0.001$; *, $P < 0.05$; ***, $P < 0.001$ (compared with Ctrl treatment); $^{\#}$, $P < 0.05$; $^{\#\#}$, $P < 0.01$; N = 16-17 per group. Data are mean ± SEM. Source data are provided as a Source Data file.

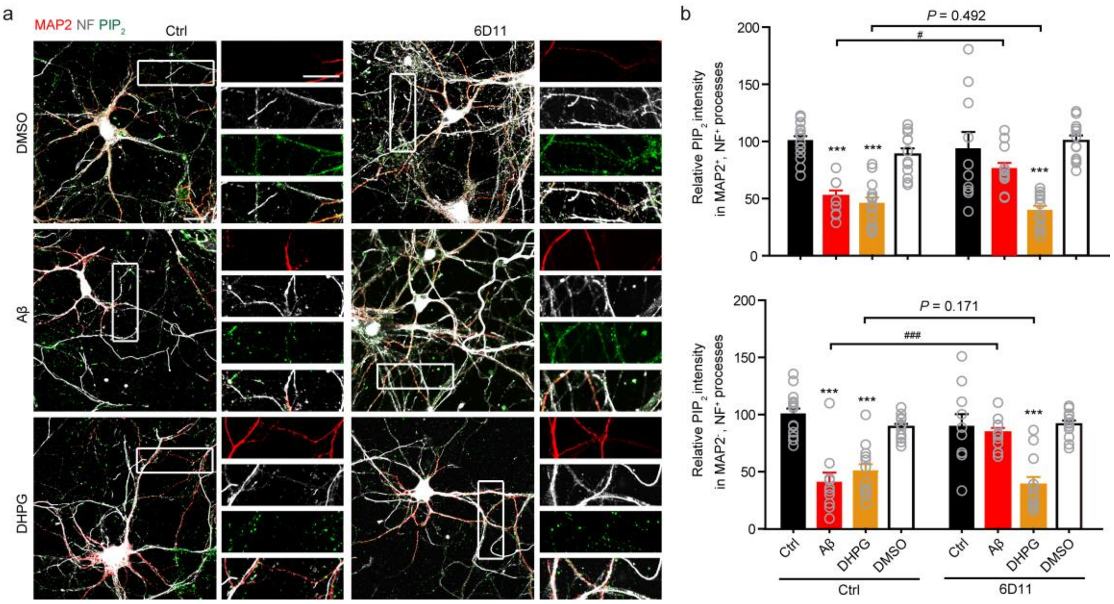

Supplementary Figure 10 Blocking PrP^{C} prevents oligomeric Aβ-induced PIP_2 reduction in cultured hippocampal neurons. **a** Confocal images of cultured hippocampal neurons blocked with medium containing anti-PrP^{C} antibody 6D11 or control (Ctrl) medium showing the effect of DMSO, oligomeric Aβ, or DHPG treatment (blank Ctrl treatment not shown) on colocalization of PIP_2, MAP2, and neurofilament (NF) along neuronal processes. Bars, 20 μm. **b** Quantification of relative PIP_2 intensity in dendrites (upper panel) and axons (bottom panel) showing 6D11 prevents Aβ-, but not DHPG-induced suppression of PIP_2 in neurites. Two-way ANOVA with post hoc Bonferroni test; upper panel: Ctrl/6D11, $F_{(1,87)} = 1.596$, $P = 0.21$; treatment, $F_{(3,87)} = 32.585$, $P < 0.001$; bottom panel: Ctrl/6D11, $F_{(1,88)} = 1.650$, $P = 0.202$; treatment, $F_{(3,88)} = 30.481$, $P < 0.001$; ***, $P < 0.001$ (compared with Ctrl-Ctrl or 6D11-Ctrl group); $^{\#}$, $P < 0.05$; $^{\#\#\#}$, $P < 0.001$; $N = 10$-14 per group. Data are mean ± SEM. Source data are provided as a Source Data file.

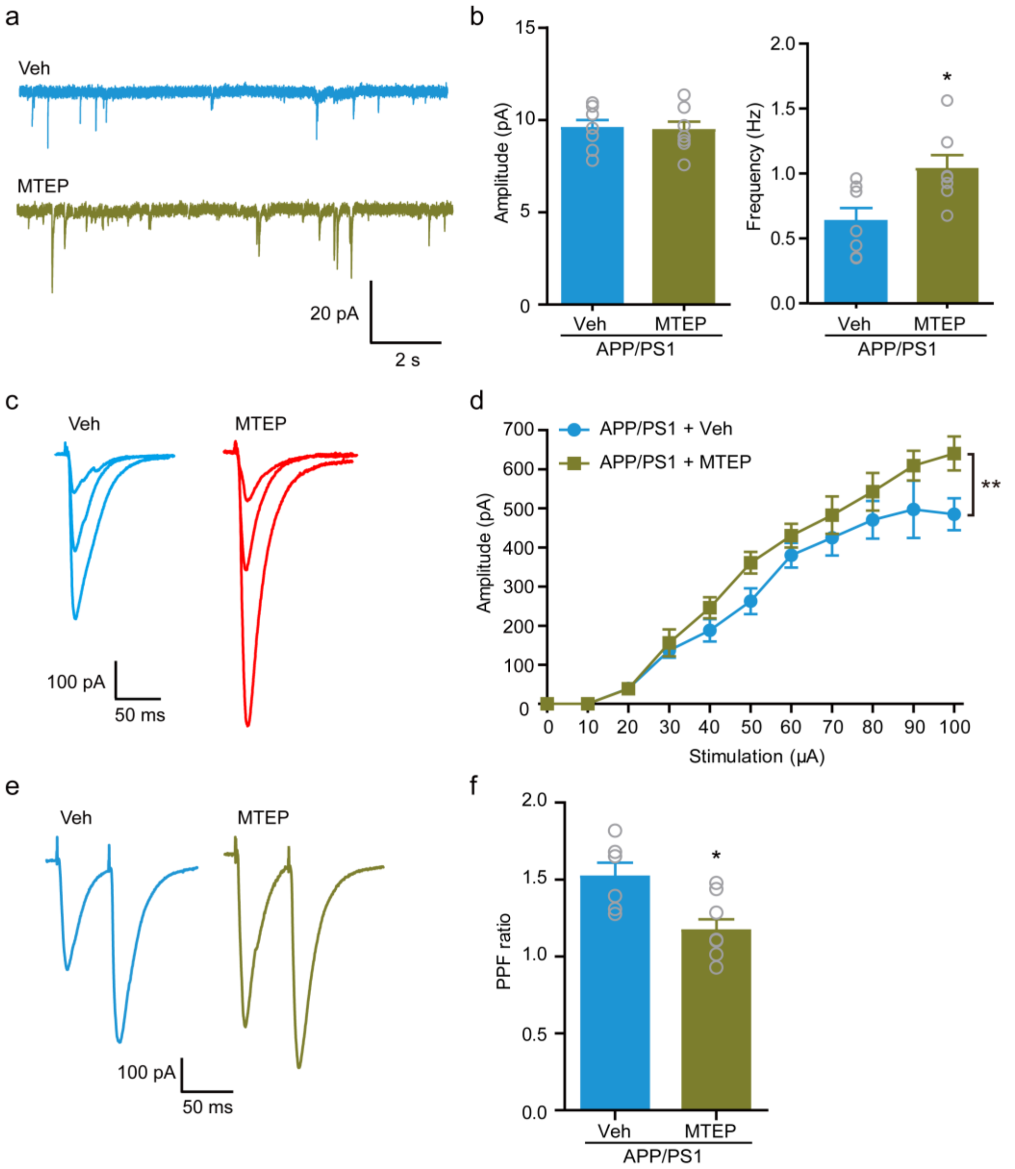

Supplementary Figure 11 Blocking mGluR5 increases excitatory synaptic transmission and decreases PPF in the hippocampus of 6-7-month-old APP/PS1 mice. **a, b** Representative traces (**a**) of mEPSCs in CA1 pyramidal neurons and quantification (**b**) of mEPSC amplitude (left) and frequency (right) in hippocampal slices of APP/PS1 mice treated with vehicle (Veh) or MTEP. *t* test; *, $P < 0.05$; $N = 7$ per group. **c, d** Representative traces of SC-CA1 EPSCs evoked by stimulus intensities of 30, 50, and 100 μA (**c**) and quantification of EPSC amplitude to stimulus intensity (**d**) in APP/PS1 hippocampal slices treated with Veh or MTEP. Two-way

ANOVA with post hoc Bonferroni test; treatment, $F_{(1,88)} = 14.648$, $P < 0.001$; stimulation, $F_{(10,88)} = 81.036$, $P < 0.001$; **, $P < 0.01$; $N = 5$ per group. **e, f** Representative traces (**e**) and quantification (**f**) of PPF of SC-CA1 EPSCs in APP/PS1 hippocampal slices treated with Veh or MTEP. *t* test; *, $P < 0.05$; $N = 6\text{-}8$ per groups. Data are mean ± SEM. Source data are provided as a Source Data file.

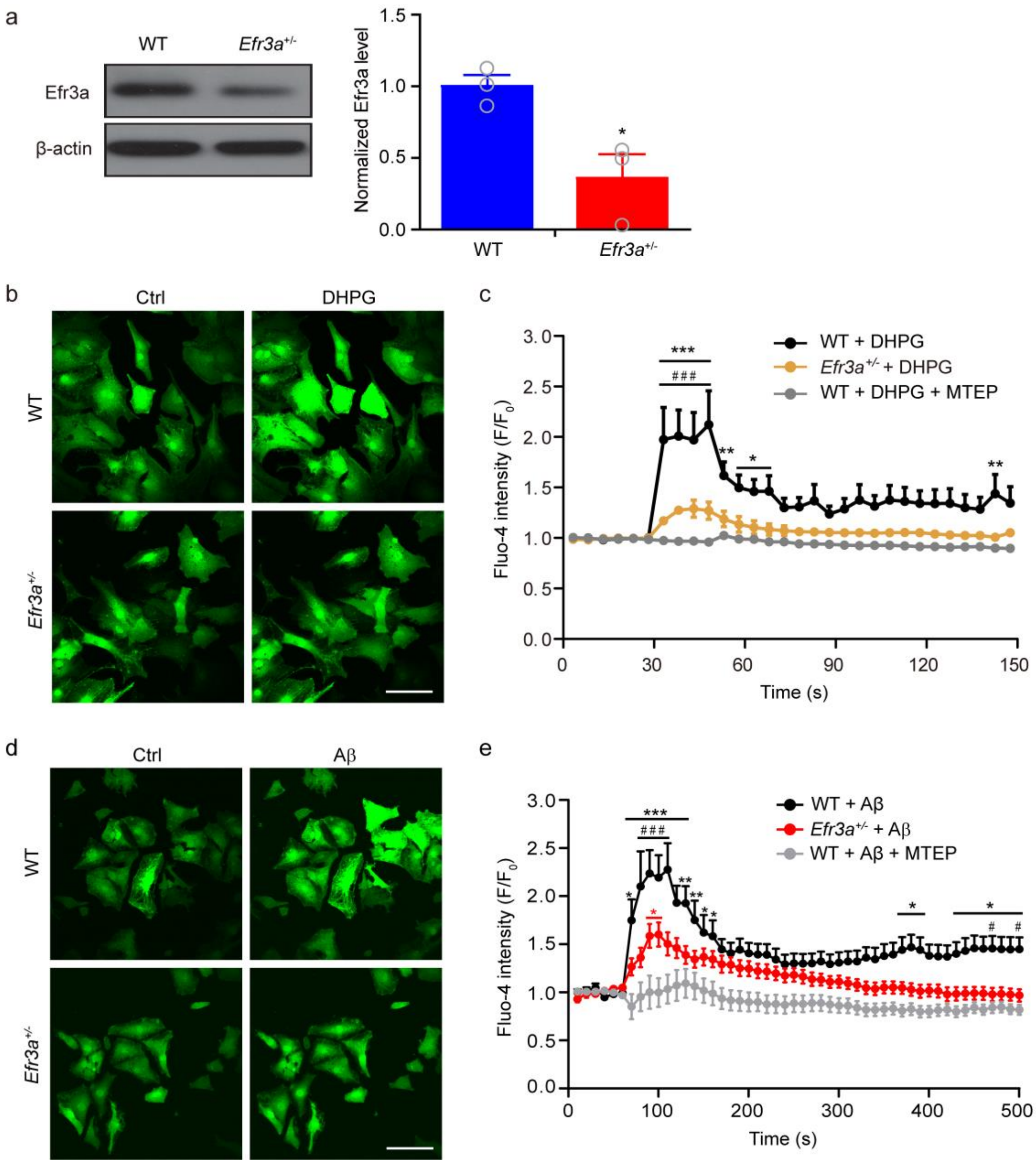

Supplementary Figure 12 DHPG- or oligomeric Aβ-induced, mGluR5-mediated increase in $[Ca^{2+}]_i$ is downregulated by halving *Efr3a* copy number in astrocytes. **a** Representative Western blots (left) and quantification (right) of Efr3a in the hippocampus from WT and *Efr3a*$^{+/-}$ mice. *t* test; *, $P < 0.05$; $N = 3$ per group. **b-e** Confocal images of cultured astrocytes from WT and *Efr3a*$^{+/-}$ mice stained with fluo-4 (**b, d**) and the time course (**c, e**) of the relative astrocytic fluo-4 intensity (F/F_0) in

response to control (Ctrl)/DHPG (**b, c**) or Ctrl/Aβ (**d, e**) treatment. MTEP blocks DHPG- (**c**) or Aβ-induced (**e**) Ca^{2+} increase in astrocytes. Bars, 50 μm. Two-way RM ANOVA with post hoc Bonferroni test; in **c**: group, $F_{(2,1769)} = 13.509$, $P < 0.001$; time, $F_{(58,1769)} = 5.354$, $P < 0.001$; in **e**: group, $F_{(2,2744)} = 11.457$, $P < 0.001$; time, $F_{(98,2744)} = 2.833$, $P < 0.001$; *, $P < 0.05$; **, $P < 0.01$; ***, $P < 0.001$ (compared with MTEP groups in **c** and **e**); $^{\#}$, $P < 0.05$; $^{\#\#\#}$, $P < 0.001$ (WT + DHPG vs. *Efr3a*$^{+/-}$ + DHPG group in **c**, WT + Aβ vs. *Efr3a*$^{+/-}$ + Aβ group in **d**); N = 12-25 per group. Data are mean ± SEM. Source data are provided as a Source Data file.

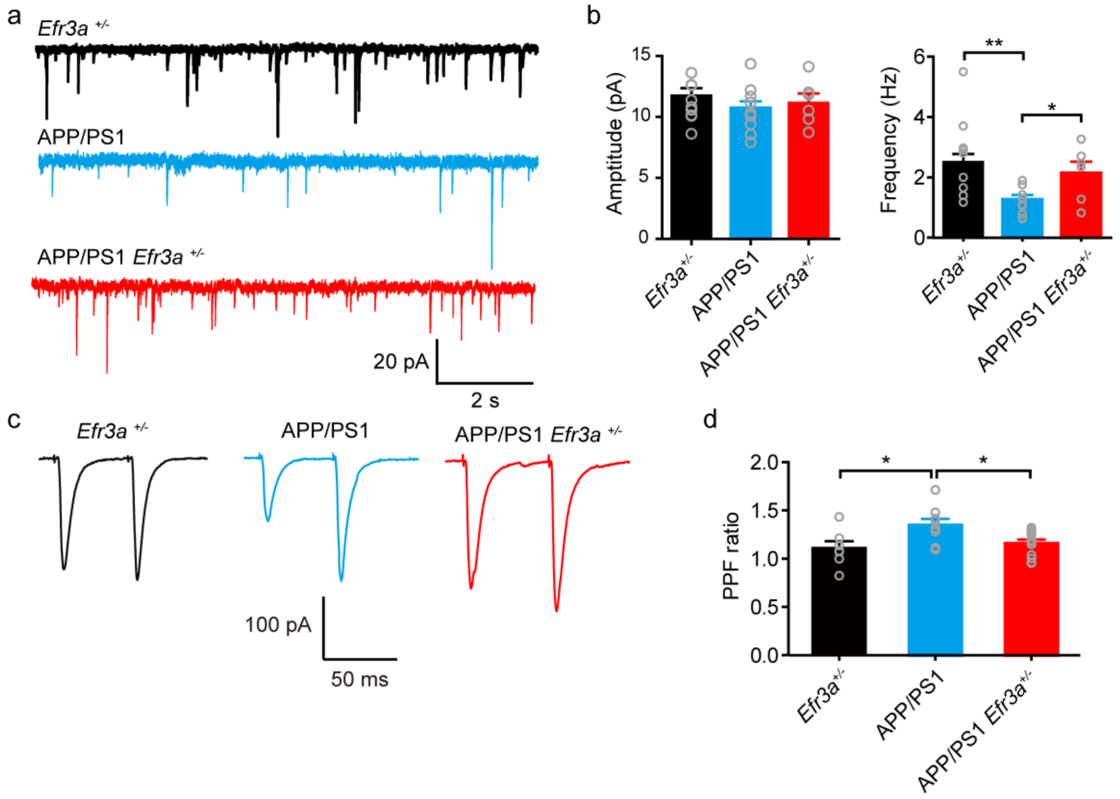

Supplementary Figure 13 Halving *Efr3a* copy number ameliorates early excitatory synaptic deficits in APP/PS1 mice. **a, b** Representative traces (**a**) of mEPSCs in CA1 pyramidal neurons and quantification (**b**) of mEPSC amplitude (left) and frequency (right) in 6-7-month-old WT, APP/PS1, and APP/PS1 *Efr3a*$^{+/-}$ mice. One-way ANOVA with post hoc Dunnett's test; $F_{(2,24)}$ = 0.17 (amplitude); $F_{(2,24)}$ = 5.78 (frequency); *, P < 0.05, **, P < 0.01; N = 6-11 per group. **c, d** Representative traces (**c**) and quantification (**d**) of PPF of SC-CA1 EPSCs in 6-7-month-old WT, APP/PS1, and APP/PS1 *Efr3a*$^{+/-}$ mice. One-way ANOVA with post hoc Dunnett's test; $F_{(2,25)}$ = 4.9; *, P < 0.05; N = 7-12 per group. Data are mean ± SEM. Source data are provided as a Source Data file.

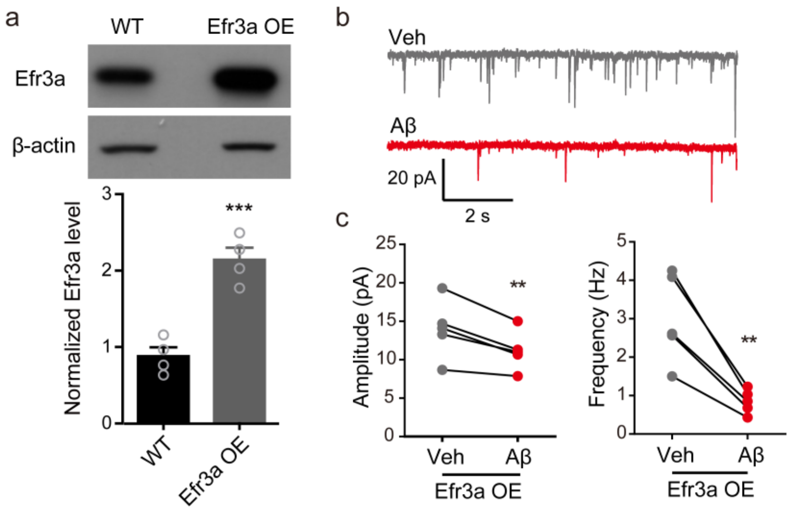

Supplementary Figure 14 Overexpression of *Efr3a* causes a more robust inhibition of mEPSC frequency in CA1 pyramidal neurons treated with oligomeric Aβ. **a** Representative Western blots of Efr3a (top) and quantification of Efr3a expression (bottom) in the hippocampus from WT and Efr3a overexpression (OE) mice. *t* test; ***, $P < 0.001$; $N = 4$ per group. **b, c** Representative traces (**b**) of mEPSCs in CA1 pyramidal neurons and quantification (**c**) of mEPSC amplitude (left) and frequency (right) in hippocampal slices from Efr3a OE mice before (Veh) and after oligomeric $A\beta_{42}$ (400 nM) treatment. *t* test; **, $P < 0.01$; $N = 5$ per group. Data are mean ± SEM. Source data are provided as a Source Data file.

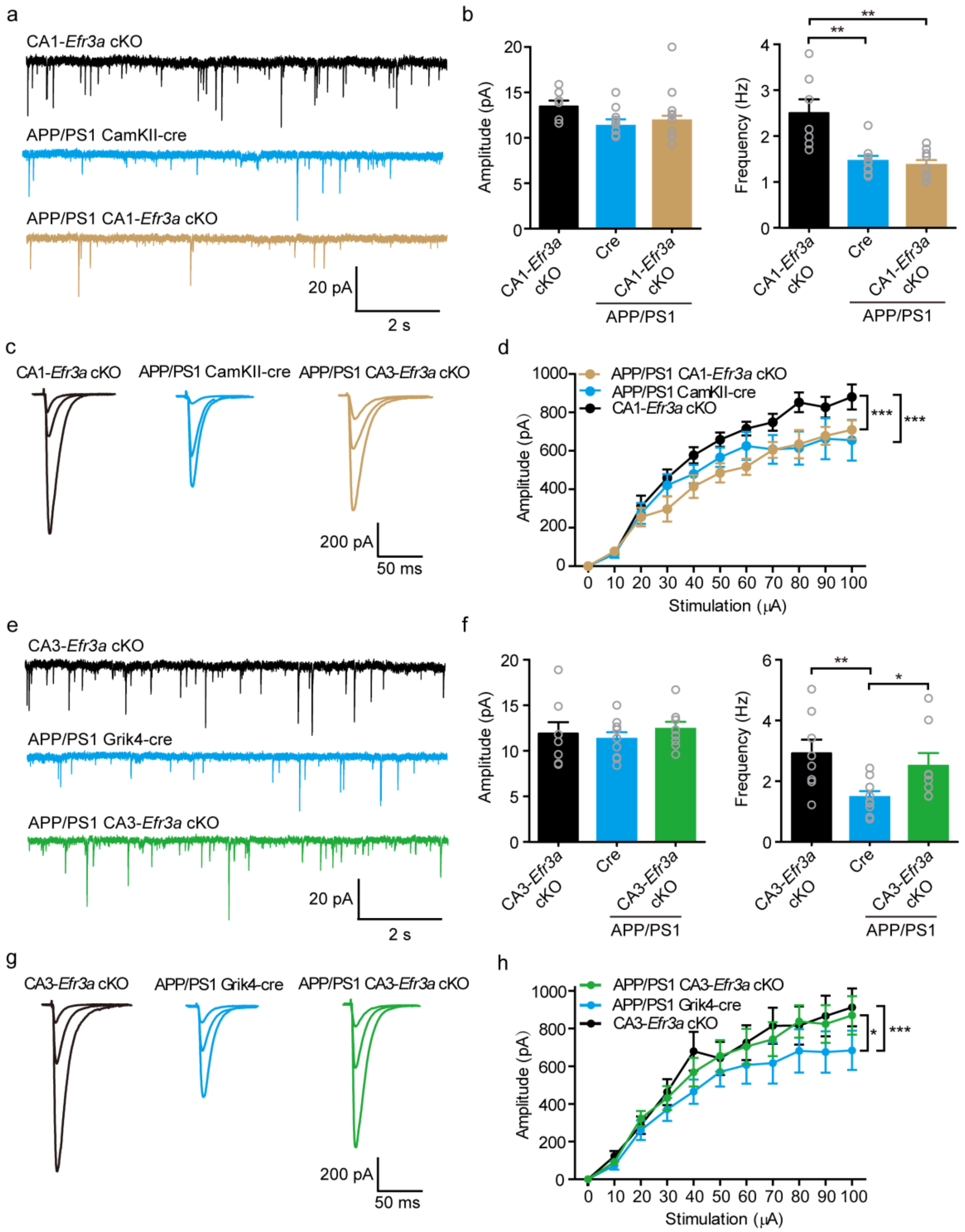

Supplementary Figure 15 Selectively knocking out *Efr3a* in CA3 area recues excitatory transmission deficits in APP/PS1 mice. **a, b** Representative traces (**a**) of mEPSCs in CA1 pyramidal neurons and quantification (**b**) of mEPSC amplitude (left) and frequency (right) in 6-7-month-old CA1-*Efr3a* cKO, APP/PS1 CamKII-cre, and

APP/PS1 CA1-*Efr3a* cKO mice. One-way ANOVA with post hoc Dunnett's test; $F_{(2,22)}$ = 0.9 (amplitude); $F_{(2,22)}$ = 11.8 (frequency); **, $P < 0.01$; N = 7-9 per group. **c, d** Representative traces of SC-CA1 EPSCs evoked by stimulus intensities of 10, 20, and 100 μA (**c**) and quantification of EPSC amplitude to stimulus intensity (**d**) in 6-7-month-old CA1-*Efr3a* cKO, APP/PS1 CamKII-cre, and APP/PS1 CA1-*Efr3a* cKO mice. Two-way ANOVA with post hoc Bonferroni test; animal, $F_{(2,176)}$ = 16.957, $P <$ 0.001; stimulation, $F_{(10,176)}$ = 68.471, $P < 0.001$; ***, $P < 0.001$; N = 6-7 per group. **e, f** Representative traces (**e**) of mEPSCs in CA1 pyramidal neurons and quantification (**f**) of mEPSC amplitude (left) and frequency (right) in 6-7-month-old CA3-*Efr3a* cKO, APP/PS1 Grik4-cre, and APP/PS1 CA3-*Efr3a* cKO mice. One-way ANOVA with post hoc Dunnett's test; $F_{(2,22)}$ = 0.35 (amplitude); $F_{(2,22)}$ = 4.32 (frequency); *, $P < 0.05$; **, $P < 0.01$; N = 8-9 per group. **g, h** Representative traces of SC-CA1 EPSCs evoked by stimulus intensities of 10, 30, and 100 μA (**g**) and quantification of EPSC amplitude to stimulus intensity (**h**) in 6-7-month-old CA3-*Efr3a* cKO, APP/PS1 Grik4-cre, and APP/PS1 CA3-*Efr3a* cKO mice. Two-way ANOVA with post hoc Bonferroni test; animal, $F_{(2,176)}$ = 7.037, P = 0.001; stimulation, $F_{(10,176)}$ = 32.706, $P < 0.001$; *, $P < 0.05$; ***, $P < 0.001$; N = 6-7 per group. Data are mean ± SEM. Source data are provided as a Source Data file.

Supplementary Table 1. The primers used in the study. TA, the optimal Annealing Temperature.

Primer name		Sequence	TA
Efr3a flox	Efr3a_LoxP1-1-F	GCAGGACCACTGTTTTGGCTGC	64°C
	Efr3a_LoxP1-1-R	AGCCAGGGATGCGACTCATGA	
Efr3a KO	Efr3a_KO-1-F	TTATTTAGTATGTTGGACGAG	56°C
	Efr3a_KO-1-R	AACATGGAGGTTAAGTTTGT	
Efr3a KI	Efr3a_OE-1-F	CACTTGTAAGGAGTGGTGAAGGACCA	64°C
	Efr3a_OE-1-R	ATTGTGCAAGGCCCTGGGCTTAAT	
Grik4-Cre	Grik4-Cre-F	GCGGTCTGGCAGTAAAAACTATC	59°C
	Grik4-Cre-R	GTGAAACAGCATTGCTGTCACTT	
CamK2a-Cre	CamK2a-Cre-F	GACAGGCAGGCCTTCTCTGAA	61°C
	CamK2a-Cre-R	CTTCTCCACACCAGCTGTGGA	
APP/PS1	PSEN1dE9-F	GTGGATAACCCCTCCCCCAGCCTAGACC	64°C
	PSEN1dE9-R	AATAGAGAACGGCAGGAGCA	
	APP-F	GACTGACCACTCGACCAGGTTCTG	64°C
	APP-R	CTTGTAAGTTGGATTCTCATATCCG	
5xFAD	oIMR 3610-F	AGGACTGACCACTCGACCAG	54°C
	oIMR 3611-R	CGGGGGTCTAGTTCTGCAT	
	oIMR 1644-F	AATAGAGAACGGCAGGAGCA	54°C

	oIMR 1645-R	GCCATGAGGGCACTAATCAT	
M146V	oIMR 1586-F	AGGCAGGAAGATCACGTGTTCAAGTAC	69°C
	oIMR 1587-R	CACACGCACACTCTGACATGCACAGGC	

Supplementary Table 2. The drugs used in the study.

**Reagents**	**Company**	**Catalogue Number**
Tetrodotoxin	Abcam	ab120054
Bicuculline	Abcam	ab120108
β-amyloid$_{42}$	Thermo fisher scientific	1764958A
Mg-ATP	Sigma-Aldrich	P9187
Tris-GTP	Sigma-Aldrich	G9002
QX-314	Abcam	ab120117
Phosphocreatine di(tris) salt	Sigma-Aldrich	P1937
Tamoxifen	Sigma-Aldrich	T5648
PI(4,5)P_2 MASS ELISA KIT	Echelon Bioscience	K-4500
HFIP	Sigma-Aldrich	52517
DMSO	Sigma-Aldrich	D8418
DNQX	Abcam	ab120018
D-AP5	Abcam	ab120003
FM 1–43	Biotium	70030
ADVASEP-7	Biotium	70029
Fluo-4	Thermo fisher scientific	14201
DHPG	Abcam	ab120007
MTEP	Abcam	ab144307
U73122	Abcam	ab120998

PI(4,5)P_2 diC8	Echelon Bioscience	P-4508
PI4P diC16	Echelon Bioscience	P-4016
PI(3,4)P_2 diC16	Echelon Bioscience	P-3416
PI(3,5)P_2 diC16	Echelon Bioscience	P-3516
PI(3,4,5)P_3 diC16	Echelon Bioscience	P-3916
Sunflower oil	Sigma-Aldrich	S5007
Acrylamide	Shenggong (China)	A1032
N,N'-MethyleneBisacrylamide	Sigma-Aldrich	M7279
West Pico	Pierce	34078
West Femto	Pierce	34095
Ammonium Persulfate	Sigma-Aldrich	A3678
TEMED	Sigma-Aldrich	T9281
Glycine	Biosharp (China)	56-40-6
SDS	Sigma-Aldrich	L4390
Tris-Base	Sigma-Aldrich	T1503
Albumin bovine V (BSA)	BIOSHARP	Amresco0332
Skim milk powder	BIOFROX	1172GR100
Albumin from chicken egg white powder (Ovalbumin)	Sigma-Aldrich	A5253
Gelatin	Shenggong (China)	G9764
Neurobasal medium	Thermo fisher scientific	21103-049

Fetal bovine serum (FBS)	Thermo fisher scientific	10099-141
GlutaMax™	Thermo fisher scientific	35050-061
LY-411575	Sigma-Aldrich	SML0506

Supplementary methods

Animals

All procedures were carried out in accordance with the National Institutes of Health Guidelines for the Care and Use of Laboratory Animals and were approved by the Animal Advisory Committee at Zhejiang University. *Efr3a* double-flox (*Efr3a*$^{f/f}$) mice were generated as reported by Qian et al.[1] , and *Efr3a*$^{+/-}$ heterozygotes were generated by breeding *Efr3a*$^{f/f}$ mice to EIIa-cre mice as described in a previous report[2]. B6 (stock number 000664), APP/PS1 double-transgenic [B6.Cg-Tg (APPswe,PSEN1dE9)85Dbo/Mmjax, stock number 34832], 5xFAD [B6.Cg-Tg(APPSwFlLon,PSEN1*M146L*L286V)6799Vas/Mmjax, stock number 34848], PS1M146VKI [B6.129-Psen1^{tm1Mpm}/J stock number 004193], Grik4-cre [C57BL/6-Tg(Grik4-cre)G32-4Stl/J, stock number 006474], and Camk2a-creERT2 [B6;129S6-Tg(Camk2a-cre/ERT2)1Aibs/J, stock number 012362] mice were purchased from The Jackson Laboratory (Bar Harbor, ME). *Efr3a*$^{f/f}$, *Efr3a*$^{f/f}$-Grik4-cre, *Efr3a*$^{f/f}$-Camk2a-creERT2, APP/PS1-*Efr3a*$^{f/f}$-Grik4-cre, APP/PS1-Grik4-cre, APP/PS1-*Efr3a*$^{f/f}$-Camk2a-creERT2, and APP/PS1-Camk2a-creERT2 animals were obtained by heterozygous mating. The transgenic strain overexpressing Efr3a was generated with a BAC transgenic construct containing the genomic DNA of *Efr3a*

carrying the miss sense mutation of S356A. Tamoxifen was intraperitoneally (i.p.) injected once a day for 5 consecutive days at a dose of 100 mg per kg to induce cre recombinase expression in the creER lines. *Efr3a*$^{+/-}$ mice embryos for primary neuron and astrocyte cultures were obtained from mating *Efr3a*$^{+/-}$ to B6 WT mice. All mice were housed at the Animal Facility of Zhejiang University under a 12-h light/dark cycle and had access to food and water ad libitum. For behavioral experiments, only male mice were used. The mouse genotypes were identified by PCR using genomic DNA from mouse tails and embryo tissues. The primers information can be found in Supplementary Table 1.

To inhibit γ-secretase, we treated mice with LY-411575 (SML0506, Sigma-Aldrich). LY-411575 was dissolved in DMSO at 100 mg per ml, and was then emulsified in sunflower oil (S5007, Sigma-Aldrich) at 1 mg per ml. γ-secretase inhibitor treatment was carried out as previously described[3]. Briefly, mice were injected subcutaneously once daily for 2 days with 3 mg per kg LY-411575 or vehicle (sunflower oil). 8 to 10 h after the injection, mice were sacrificed for electrophysiology recording.

Antibodies and drugs

The following commercially available antibodies were used: rabbit anti-amyloid precursor protein/C-terminal fragments (anti-CTFs, A8717, Sigma-Aldrich, 1:6000), purified mouse anti-β-Amyloid, 1-16 (6E10, 803003, Biolegend; 1:1000 for Western blot; 2 μg per ml for slice recordings as previously reported[4, 5]), rabbit anti-Efr3a (HPA023402, Sigma-Aldrich, 1:1000), mouse anti-PLCβ1, D-8 (sc-5291, Santa Cruz, 1:200), mouse anti-PLCβ4, A-8 (sc-16613, Santa Cruz, 1:100), mouse anti-β-actin

(TA-09, ZSGB-BIO, 1:20000), and HRP-conjugated secondary antibodies [(Goat anti-rabbit IgG (H+L), 31460, 1:20000; goat anti-mouse IgG (H+L), 31430, 1:20000; Thermo fisher scientific); goat anti-mouse IgG (H+L), 70-GAM007, Multi Sciences, 1:5000] were used in Western blotting; mouse anti-PIP_2 antibody (ab11039, Abcam, 1:200), chicken anti-neurofilament-L (anti-NF, CH22105, Neuromics, 1:250), rabbit anti-MAP2 antibody (ab32454, Abcam, 1:8000), rabbit anti-mGluR5 (extracellular) (AGC-007, Alomone labs, 1:50), mouse anti-MAP2 (M4403, Sigma-Aldrich, 1:1000), purified anti-CD230 (Prion) antibody (6D11, 808001, Biolegend, 5 μg per ml), and Alexa Fluor-conjugated secondary antibodies [488 donkey anti-rabbit (A-21206), 546 donkey anti-mouse (A-10036), 546 donkey anti-rabbit (A-10040) (all from Thermo fisher scientific, 1:1000), 405 goat anti-rabbit IgG H&L (ab175654, Abcam, 1:500), 488 goat anti-mouse IgM mu chain (ab150121, Abcam, 1:500), and 647 goat anti-chicken IgY H&L (ab150175, Abcam, 1:1000)] were used in immunocytochemistry.

To prepare oligomeric $A\beta_{42}$[6], the $A\beta_{42}$ lyophilized powder (03112, Thermo Fisher Scientific) was first suspended in 100% 1,1,1,3,3,3-hexafluoro-2-propanol (HFIP, 52517, Sigma-Aldrich) at a concentration of 1 mM. The $A\beta_{42}$-HFIP solution was then incubated in polypropylene vials for complete solubilization at room temperature (RT) for 2 h. HFIP was allowed to evaporate under a slight stream of nitrogen until a clear peptide film was observed at the bottom of the vials. The vials were stored at -80°C until use. Twelve hours before experiments, the film was re-suspended by adding DMSO (D8418, Sigma-Aldrich) at a concentration of 5 mM and sonicated at RT for 10

min. The Aβ_{42}-DMSO solution was diluted 12 times in sterile PBS or culture medium and incubated at 4°C for 12 h. Following a 5 min centrifugation at 14,000 g, the concentration of the supernatant (~ 100 μg per ml) was determined by a microplate reader (SpectraMax 190, Molecular Devices) and the oligomeric Aβ_{42} solution was diluted to 400 nM (based on monomeric Aβ_{42}) accordingly. To prepare fibrillar Aβ_{42}, the Aβ_{42}-DMSO solution was diluted 12 times in 10 mM HCl and incubated for 24 h at 37°C.

Tamoxifen (T5648, Sigma-Aldrich) was dissolved in 100% ethanol at 100 mg per ml, and then was emulsified in sunflower oil (S5007, Sigma-Aldrich) at 10 mg per ml and vortexed for 5-10 min until the solution was clear. The stock solution was aliquoted and stored at -20°C. For slice recordings, PI(4,5)P_2 diC8 (PIP_2, P-4508, Echelon Bioscience) was dissolved in the electrode solution at final concentration of 200 μM. For ELISA assay, PI(4,5)P_2 diC8, PI4P diC16 (P4016), PI(3,4)P_2 diC16 (P-3416), PI(3,5)P_2 diC16 (P-3516), and PI(3,4,5)P_3 diC16 (P3916; all from Echelon Bioscience) were dissolved in PBS containing 0.25% Protein Stabilizer (PBS 0.25%PS) at final concentrations of 10, 50, and 250 pM. DHPG (ab120007, Abcam) was dissolved in the bath solution or culture medium and applied at a final concentration of 50 μM. MTEP (ab144307, Abcam) and U73122 (ab120998, Abcam) were dissolved in DMSO and applied to the bath solution or culture medium at a final concentration of 10 μM. In the Ca^{2+} imaging experiment, DHPG was applied at a final concentration of 100 μM. Drugs were added in the ACSF perfusate in slice recording experiments and in the culture medium 24 h before various experiments performed on

cell cultures. The final concentrations of DMSO did not exceed 0.1% throughout the study. Drugs used in the study are described in Supplementary Table 2.

Golgi staining

Golgi stainings were carried out using an FD Rapid GolgiStain Kit (PK401, FD NeuroTechnologies) according to the manufacturer's instructions[7]. In brief, nonperfused mouse (WT and APP/PS1; 6-7-month-old) brains were immersed in impregnation solution for 2 weeks, and then transferred to “Solution C” for 2 days. Sections of 200 μm thickness were serially cut with a freezing microtome (CM30503, Leica). Sections were mounted on 3% gelatin-coated slides and allowed to dry for 2 weeks before being stained with silver nitrate solution “Solution D and E”, dehydrated through descending alcohol series, and mounted with Permount. Images were acquired with an Olympus BX53 microscope at RT, and neuronal morphology analysis was performed using the NIH ImageJ software. At least 5 pyramidal neurons in the hippocampal CA1 region per mouse were randomly selected. The density of dendritic spines of a distinct branch was measured by a 100X oil-immersion objective (numerical aperture 1.3). Spines visible along both sides of dendritic segments were counted and expressed as mean number of spines per micrometer. Student t-test was used to determine significant levels between the WT and APP/PS1 groups.

Slice recording

Hippocampal brain slices were prepared from 4- or 6-7-month-old mice. For whole-cell recordings, mice were anesthetized with isoflurane and brains were dissected rapidly and immersed in ice-cold and oxygenated (95% O_2 / 5% CO_2) ACSF

(in mM: 124 NaCl, 2 KCl, 2 $MgSO_4$, 1.25 KH_2PO_4, 2 $CaCl_2$, 26 $NaHCO_3$, 10 D-glucose, pH 7.4, 300 mOsm). Transverse slices of hippocampus (300 μm) were cut with a tissue slicer (VT 1200S, Leica) in oxygenated ACSF. Slices were allowed to recover ~12 min in ACSF with low Na^+ and Ca^{2+} concentrations (in mM: 110 N-methyl-D-glucamine, 110 HCl, 2.5 KCl, 1.2 NH_2PO_4, 10 $MgSO_4$, 0.5 $CaCl_2$, 25 $NaHCO_3$, 25 D-glucose, pH 7.4, 300 mOsm) at 32°C, and subsequently in normal ACSF for 1 h at RT. For fEPSP recordings, mouse brains were dissected rapidly and immersed in ice-cold and oxygenated cutting solutions (in mM: 234 Sucrose, 5 KCl, 1.25 NaH_2PO_4, 5 $MgSO_4$, 26 $NaHCO_3$, 25 Dextrose, 1 $CaCl_2$, balanced with 95% O_2 / 5% O_2). Transverse brain slices (350 μm) containing the hippocampus were incubated in oxygenated ACSF and were allowed to recover ~25 min in ACSF at 32°C, and subsequently for ≧1 h at RT.

Whole-cell recordings were performed as previously described[7, 8]. Hippocampal slices were transferred to the recording chamber at 32°C and perfused continuously with ACSF bubbled with 95% O_2 / 5% CO_2 to ensure adequate oxygenation of slices. CA1 pyramidal neurons were identified under infrared differential interference contrast (IR-DIC) optics based on their location and morphology. Borosilicate glass (Sutter instruments) pipettes (3 - 5 MΩ) were pulled with a horizontal pipette puller (P97, Sutter instruments) and were filled with artificial intracellular fluid (in mM: 100 $CsCH_3SO_3$, 20 KCl, 10 HEPES, 4 Mg-ATP, 0.3 Tris-GTP, 7 $Tris_2$-Phosphocreatine, 3 QX-314; pH 7.3, 285-290 mOsm). Pipettes were connected to the headstage of a Heka EPC 10 amplifier (Heka Elektronik), and fast and slow capacitances as well as

series resistance compensations were carefully adjusted. Liquid junction potentials were not corrected. mEPSC signals were recorded at -70 mV in ACSF containing 0.5 μM tetrodotoxin (TTX; Abcam) and 10 μM bicuculline (Abcam). Series resistance was normally less than 20 MΩ and recordings exceeding 20% change in series resistance were terminated and discarded. Recordings were filtered at 2.0 kHz and digitized at 10 kHz.

Evoked EPSCs were elicited in the presence of 10 μM bicuculline using a bipolar stimulating electrode (CE2C75, FHC Inc.) placed in stratum radiatum 300 μm away from the recording site. The rectangle current pulses (duration: 180 μs, frequency: 0.1 Hz) were delivered via a constant-current stimulator (SIU91A, Cygnus Technology). PPF experiments were carried out by delivering a pair of stimuli with an interval of 50 ms. PPF was assessed by the paired-pulse ratio (the second EPSC amplitude / the first EPSC amplitude). To estimate the RRP size and release probability, a repeated (10-20 repeats, 0.033 Hz) 20 Hz train stimulation (40 stimuli) protocol was used to evoke 40 EPSCs. To effectively discharge the RRP, a slightly higher stimulation intensity than the minimal stimulation was used to give about 5% failures. The RRP size was calculated by linear interpolating the linear portion of the cumulative EPSC amplitude plot to virtual stimulus 0. The release probability was calculated as the mean amplitude of the 1st EPSC during the repeated train stimulations divided by the RRP size.

Field EPSPs were elicited by stimulating the SC and recording with a borosilicate glass electrode filled with ACSF placed in CA1 stratum radiatum. Baseline and tetanic

stimulations were delivered by a bipolar stimulating electrode (CE2C75, FHC Inc.) placed 200 - 300 μm away from the recording electrode. To record baseline responses before LTP induction, the intensity of each stimulus was adjusted to evoke fEPSPs with an amplitude 30 - 50% of the maximum. We also adjusted the amplitude of the baseline fEPSPs in control groups to match that in groups with reduced fEPSPs. Baseline fEPSPs were evoked at 0.05 Hz and recorded for at least 20 min (response variability < 10%). Three bursts of 20 pulses at 100 Hz separated by 1.5 s were delivered to induce LTP as previously described[7]. We recorded LTP in at least 7 slices from 3 - 4 mice for each group. Field EPSPs were recorded, filtered (1 kHz), and sampled (20 kHz) by a Heka EPC 10 amplifier.

To investigate synthetic Aβ-induced alterations in mEPSCs, evoked EPSCs, and short- and long-term plasticity, we perfused hippocampal slices with Aβ-containing ACSF for at least 20 min before acquiring data, unless the time course data were taken.

Cell culture

Primary hippocampal neuron cultures were prepared from embryonic day 18 (E18) mice[7]. Briefly, embryos were removed from maternal mice anesthetized with isoflurane and euthanized by decapitation. Hippocampi were dissected and placed in Ca^{2+}- and Mg^{2+}-free HEPES-buffered Hank's balanced salt solution (HBSS; pH 7.45), followed by a digestion with 0.25% w/v trypsin. After trituration through a Pasteur pipette, neurons were centrifuged (1000 g for 5 min) and resuspended in Neurobasal medium containing 2% B27 serum-free supplement, 1% v/v penicillin/streptomycin

(P/S), 0.5 mM glutamine, and 10 μM glutamate (Sigma-Aldrich). Dissociated cells were then plated at a density of 0.03 - 0.05 x 10^6 cells per cm^2 onto 12 mm round coverslips in 24-well plates (Corning Costar®, for lentivirus infection and immunofluorescence staining), glass bottom confocal dishes (801002, NEST Biotechnology, for vesicle detection) or 6-well plates (Corning Costar®, for Western blotting and ELISA assay) pre-coated with poly-D-lysine (PDL, 50 μg per ml; Sigma-Aldrich). Cultures were kept at 37°C in a 5% v/v CO_2 humidified incubator. Thereafter, one third to half of the medium was replaced twice a week with Neurobasal culture medium containing 2% B27 supplement and 0.5 mM glutamine.

Single-cell micro-island cultures of hippocampal neurons were prepared from E18 mice[7]. Briefly, 6.5 mm Transwell® inserts (pore size 0.4 μm; BD Biosciences) in 24 well plates were coated with PDL (12 h before culture), and coverslips were sprayed with island substrate solution containing 1 mg per ml PDL and 3 mg per ml rat tail collagen (A1048301, Thermo Fisher Scientific) using a presterilized glass atomizer (3 h before culture). Dissociated cells for micro-island cultures were similarly prepared as dissociated cells for primary hippocampal neuron cultures. Dissociated cells were then plated at a density of 2000 cells per cm^2 onto coverslips in 24-well plates (for micro-island culture) or at a density of 50000 cells per cm^2 in Transwell® inserts in 24-well plates (as high density neuronal feeder layer). After an adherence time of 4 h, the transwell inserts with neurons (high density) were placed into 24-well plates with neurons on coverslips (low density). The low density system can facilitate the survival of sparse individual neurons grown in islands of PDL substrates and thus the

formation of autaptic connections. In addition, co-culture with high density neurons may allow low density neurons to receive trophic support that is sufficient to enable long term survival. Cultures were kept at 37°C in a 5% v/v CO_2 humidified incubator. Thereafter, one third to half of the medium was replaced every five days with Neurobasal culture medium containing 2% B27 supplement and 0.5 mM glutamine.

Astrocyte cultures were prepared from 0-1-day-old (P0-1) mice[7]. Cortices were dissected from 0-1-day-old mice and digested with 0.25% w/v trypsin in DMEM. Tissue was triturated and resuspended after centrifugation in astrocyte culture medium [DMEM containing 10% v/v fetal bovine serum (FBS), 1% v/v P/S]. Cells were plated in T-75 flasks at a density of 2 cortices per flask (Corning Costar®) pre-coated with PDL. Cells were grown for at least 7 days at 37°C with 5% v/v CO_2, and a complete medium change was performed every other day. At confluence after DIV8-10, cultures were shaken for 12-16 h at 250 g at 37°C on an orbital shaker (KS4000i Control Incubating Shaker, IKA) followed by an incubation in culture medium containing 20 μM cytosine-1-β-D-arabinofuranosid (Sigma-Aldrich) for 2-3 days to deplete the precursor cells and to achieve a confluent layer of astrocytes. Astrocytes were then subcultured in PDL-coated glass bottom confocal dishes via enzymatic digestion with 0.05% w/v trypsin with EDTA for Ca^{2+} imaging experiments.

FM1-43 loading and synaptic vesicle detection

Cultured neurons (DIV14) were transferred into a standard bath solution containing (in mM): 145 NaCl, 5 KCl, 2 $CaCl_2$, 2 $MgCl_2$, 10 glucose and 10 HEPES. We also added 10 μM DNQX (Abcam) and 40 μM D-AP5 (Abcam) to the bath to inhibit AMPA and

NMDA receptors. Neurons were then incubated with 5 μM FM1-43 (70030, Biotium) in a hyperkalemic bath solution (in mM: 31.5 NaCl, 90 KCl, 2 $CaCl_2$, 2 $MgCl_2$, 25 HEPES and 30 glucose) for 90 s. Neurons were then perfused with the normal bath solution for 10 min followed by adding ADVASEP-7 (1 mM, 70029, Biotium) for 60 s to reduce background fluorescence. After an additional 10 min wash with the normal bath solution, images were taken by a confocal laser-scanning microscope (Nikon A1). FM1-43-loaded vesicles were viewed through a 40X oil-immersion objective (numerical aperture 1.3) and images were acquired at a resolution of 1024 X 1024 pixel at RT.

Western blotting

Hippocampi were obtained and homogenized using a chilled Vibrahomogenizer (Vibra cell, SONICS) in 2 ml of RIPA buffer [1% Triton X-100, 0.1% SDS, 150 mM NaCl, 2 mM EDTA, 50 mM NaF, 10 mM sodium pyrophosphate, 1.0 mM Na_3VO_4, 1.0 mM PMSF, and complete protease inhibitor cocktail (Roche)]. The lysate was then centrifuged at 20,000 g for 20 min at 4°C and the supernatant collected for Western blot analysis. The protein concentration of the probes was determined using the Pierce™ BCA Protein Assay Kit (Thermo Fisher Scientific) and the tubes were stored at -20°C. Proteins were separated on SDS-PAGE under denaturing conditions (for Efr3a, 10-15% Mini-PROTEAN TGX Gels, Bio-Rad; for Aβ and CTFs, 16.5% Tris-Tricine Gels, WSHT Biotech Inc.) and transferred to polyvinylidene fluoride (PVDF) microporous membrane (Millipore). The membranes were then blocked with 5% skim milk-TBS (for Aβ) or 0.35% gelatin-TBST (for other proteins) at RT for 1.5 h

and incubated with the primary antibodies over night at 4°C followed by HRP-conjugated secondary antibodies (Thermo Fisher Scientific) at RT for 1.5 h. Protein bands were then visualized using the ECL Western blotting detection substrate (Thermo Fisher Scientific). Densitometric analyses were determined using ImageJ software and normalized to β-actin.

Cultured neuron recording

Single-cell micro-island neuron cultures at DIV14 were transferred to a chamber perfused with the standard bath solution containing (in mM): 145 NaCl, 5 KCl, 2 $CaCl_2$, 2 $MgCl_2$, 10 glucose and 10 HEPES (pH 7.40, 290 - 310 mOsm). Neurons were recorded with patch pipettes (4 - 6 MΩ) filled with artificial intracellular fluid (in mM: 100 $CsCH_3SO_3$, 20 KCl, 10 HEPES, 4 Mg-ATP, 0.3 Tris-GTP, 7 $Tris_2$-Phosphocreatine, 3 QX-314; pH 7.3, 285-290 mOsm). Neurons were voltage clamped at -70 mV with a Heka EPC 10 amplifier and mEACs were recorded at 32°C in bath solution containing 0.5 μM TTX and 10 μM bicuculline. Individual events were counted and analyzed with MiniAnalysis software.

Lipid strip assay

PIP Strips ™ membranes (P23751, Thermo Fisher Scientific) were blocked with 0.1% ovalbumin (albumin from chicken egg white powder, A5253, Sigma-Aldrich) in TBST for 1 h at RT, and then incubated with 50 ng per ml anti-PIP_2 antibody in 0.1% ovalbumin in TBST at RT for 1 h. After being washed with TBST for three times, HRP-conjugated secondary antibody was added at RT for 1 h. Protein bands were then visualized using the ECL western blotting detection substrate (Thermo Fisher

Scientific).

ELISA PIP_2 assay

Mass ELISA Kit K-4500 from Echelon Biosciences was used to determine PIP_2 levels in hippocampi from WT and APP/PS1 mice (6-7-month-old) and in primary cultured hippocampal neurons from WT and *Efr3a*$^{+/-}$ mice[9]. Briefly, hippocampi were dissected and cut into small pieces using a pair of ophthalmic scissors, and were then transferred into a 1.5 ml centrifuge tube containing 1 ml ice-cold 0.5 mM trichloroacetic acid (TCA) immediately. Cultured neurons were incubated in TCA on ice for 5 min after the medium was carefully aspirated, and then were collected and transferred into a 1.5 ml centrifuge tube. The tubes were then centrifuged at 3000 g for 7 min at 4°C. The pallet was resuspended in 1 ml 5% TCA/1 mM EDTA solution and vortexed for 30 s. After centrifuging twice at 3000 g for 5min, the pallet was resuspended in the MeOH: $CHCl_3$: 12 N HCl (80:40:1; 750 µl) solution and vortexed for 25 min at RT to extract lipids. After centrifuging again at 3000 g for 5 min, the supernatant was transferred to a 2 ml centrifuge tube and 250 µl $CHCl_3$ and 450 µl 0.1 mM HCl were added. After vortexing for 30 s, the organic and aqueous phases were separated by centrifuging at 3000 g for 5min. The organic (lower) phase (0.5 ml) was collected and transferred into a 1.5 ml centrifuge tube and dried under a slight stream of nitrogen. PIP_2 and other PIPs [PI4P, PI(3,4)P_2, PI(3,5)P_2, and PI(3,4,5)P_3] were then detected according to the manufacturer's instructions.

Immunocytochemistry

Immunofluorescence staining was carried out in cultured neurons at DIV14 (or DIV12

for lentivirus-infected neurons)[7]. Briefly, neurons were fixed by 4% paraformaldehyde in PBS for 15 min and permeabilized by 0.2% Triton X-100 for 10 min. Neurons were blocked with 10% BSA in PBS for 2.5 h at RT, and then incubated with mouse anti-PIP_2 antibody in PBS containing 3% BSA for 1 h. After being washed with PBS for three times, fluorescent secondary antibody (Alexa Fluor 488 goat anti-mouse IgM mu chain) was added for 1 h. After being washed with PBS three times, neurons were then blocked again with 10% BSA for 1.5 h and then were incubated with rabbit anti-MAP2 and chicken anti-NF antibodies for 1 h. After being washed again with PBS for three times, fluorescent secondary antibodies [Alexa Fluor 546 anti-rabbit IgG (or Alexa Fluor 405 anti-rabbit IgG for infected neurons) and 647 anti-chicken IgY H&L] were added for 1 h. Neurons were mounted with mounting reagent for subsequent fluorescent image acquisition after being washed with PBS to remove unbound secondary antibodies. For co-staining with mGluR5, neurons were permeabilized before the second blocking step, and the following antibodies were used: mouse anti-MAP2, chicken anti-NF, rabbit anti-mGluR5, Alexa Fluor 546 anti-mouse, 647 anti-chicken IgY H&L, and 488 anti-rabbit IgG. Fluorescent images were acquired through a 60X oil-immersion objective (numerical aperture 1.4) using a Nikon A1 confocal laser-scanning microscope. Gain, threshold, and black levels were not subjected to change during individual experiments. Neuronal images were analyzed using MetaMorph with customized filter sets. All image analysis was done blind to the experimental condition.

Lentivirus-shRNA infection

To knock down PLCβ1 or PLCβ4 in primary hippocampal neurons, we infected hippocampal neuron cultures with lentiviruses carrying DsRed-PLCβ1-shRNA (PLCβ1-shRNA), DsRed-PLCβ4-shRNA (PLCβ4-shRNA), or DsRed-scramble-shRNA (Ctrl-shRNA) that were chemically synthesized by Obio Technology (Shanghai) Corp., Ltd. Target sequences for PLCβ1, PLCβ4 and Ctrl siRNAs are GCTGTCTTTGTCTACATAGAA (GenBank accession number: NM_019677), GCGACAAATGAGCCGCATT (GenBank accession number: NM_013829), and TTCTCCGAACGTGTCACGT, respectively. Hippocampal neurons at DIV7 were infected with lentiviruses according to the vendor's protocol. Neurons were then treated with Aβ or DMSO 72 h after lentiviral infection, and were subsequently used for Western blotting (interference efficiency detection) or immunofluorescence staining.

Ca^{2+} imaging

Changes in $[Ca^{2+}]_i$ were measured in cultured astrocytes from WT and *Efr3a*$^{+/-}$ mice using the calcium-sensitive fluorescent dye Fluo-4 (14201, Thermo Fisher Scientific). Astrocyte cultures were washed with Krebs buffer (in mM: 118 NaCl, 4.7 KCl, 4 $NaHCO_3$, 1.2 $MgSO_4$, 1.2 KH_2PO_4, 8.5 HEPES, 1.3 $CaCl_2$, 11.7 glucose, pH 7.4) and incubated with 4 μM Fluo-4 in Krebs buffer first at 37°C for 15 min, then at RT for 15 min. Astrocytes loaded with Fluo-4 were excited at 488 nm and fluorescence emission was detected at 525 nm. The images were taken through a 60X oil-immersion objective (numerical aperture 1.4) by a Nikon A1 confocal laser-scanning microscope. After baseline data (F_0) were taken, we recorded fluorescent signals (F) in the

presence of 100 μM DHPG or 400 nM oligomeric Aβ. The relative Fluo-4 fluorescent signals expressed in arbitrary units (F/F_0) were analyzed for individual cells using MetaMorph with a fixed set of parameters.

MWM test

The MWM tests were performed in a circular tank (120 cm in diameter and 60 cm in height) filled with opaque water at 25°C. The tank was divided into four quadrants with different navigation landmarks for each quadrant. 24 h before the acquisition test, a visible platform task was performed by measuring the time spent to find a colorful flag placed on the top of a platform in a quadrant. The visible platform task was tested in each quadrant to avoid habituation. In the hidden platform acquisition test, mice were allowed to swim freely to search for the escape platform within 60 s. The platform location remained constant throughout the test. The time taken to reach the platform was recorded as the escape latency. The mouse was allowed to stay on the platform for 10 s after the hidden platform was found. If a mouse failed to find the platform within 60 s, the mouse was guided to the platform and stayed on the platform for 10 s, and the escape latency was recorded as 60 s for this trial. The same animal was then released from a new insertion point 4 min after the previous trial. The experiment was repeated 4 times per mouse each day for 5 days. The four animal insertion points were chosen to maintain a constant distance to the platform. The mean escape latency was calculated to evaluate the spatial learning ability. 24 h after the hidden platform acquisition test, probe trials were conducted by removing the platform. Mice were placed in the diagonal quadrant of the hidden platform originally located and

were allowed to swim freely in the pool for 60 s. The numbers of entries into the area where the original platform was located and crossings over the original platform were recorded. The data were analyzed by the WaterMaze Software (Actimetrics, INC.).

References

1. Qian, Q. et al. Brain-specific ablation of Efr3a promotes adult hippocampal neurogenesis via the brain-derived neurotrophic factor pathway. *FASEB J.* **31**, 2104-2113 (2017).
2. Hu, H. et al. Efr3a Insufficiency Attenuates the Degeneration of Spiral Ganglion Neurons after Hair Cell Loss. *Front. Mol. Neurosci.* **10**, 86 (2017).
3. Harris, J.A. et al. Transsynaptic progression of amyloid-beta-induced neuronal dysfunction within the entorhinal-hippocampal network. *Neuron* **68**, 428-441 (2010).
4. Klyubin, I. et al. Amyloid beta protein immunotherapy neutralizes Abeta oligomers that disrupt synaptic plasticity in vivo. *Nat. Med.* **11**, 556-561 (2005).
5. Tampellini, D. et al. Internalized antibodies to the Abeta domain of APP reduce neuronal Abeta and protect against synaptic alterations. *J. Biol. Chem.* **282**, 18895-18906 (2007).
6. Lauren, J., Gimbel, D.A., Nygaard, H.B., Gilbert, J.W. & Strittmatter, S.M. Cellular prion protein mediates impairment of synaptic plasticity by amyloid-beta oligomers. *Nature* **457**, 1128-1132 (2009).
7. Shen, Y. et al. Postnatal activation of TLR4 in astrocytes promotes excitatory synaptogenesis in hippocampal neurons. *J. Cell. Biol.* **215**, 719-734 (2016).
8. Zhou, Y.D. et al. Arrested maturation of excitatory synapses in autosomal dominant lateral temporal lobe epilepsy. *Nat. Med.* **15**, 1208-1214 (2009).
9. Trovo, L. et al. Low hippocampal PI(4,5)P(2) contributes to reduced cognition in old mice as a result of loss of MARCKS. *Nat. Neurosci.* **16**, 449-455 (2013).
