# Supplementary material for: Amyloid β oligomers suppress excitatory transmitter release via presynaptic depletion of phosphatidylinositol-4,5-bisphosphate
Source: Nat Commun. 2019 Mar 13;10:1193. doi: 10.1038/s41467-019-09114-z (PMC6416269; doi:10.1038/s41467-019-09114-z)
Supplement: Supplementary file 3 — Reporting Summary [file 41467_2019_9114_MOESM3_ESM.pdf]

nature research

Corresponding author(s): Fude Huang, Yi Shen and Yu-Dong Zhou

Reporting Summary

Nature Research wishes to improve the reproducibility of the work that we publish. This form provides structure for consistency and transparency in reporting. For further information on Nature Research policies, see Authors & Referees and the Editorial Policy Checklist.

Statistical parameters

When statistical analyses are reported, confirm that the following items are present in the relevant location (e.g. figure legend, table legend, main text, or Methods section).

n/a	Confirmed	
☐	☒	The exact sample size (n) for each experimental group/condition, given as a discrete number and unit of measurement
☐	☒	An indication of whether measurements were taken from distinct samples or whether the same sample was measured repeatedly
☐	☒	The statistical test(s) used AND whether they are one- or two-sided<br>*Only common tests should be described solely by name; describe more complex techniques in the Methods section.*
☒	☐	A description of all covariates tested
☐	☒	A description of any assumptions or corrections, such as tests of normality and adjustment for multiple comparisons
☐	☒	A full description of the statistics including central tendency (e.g. means) or other basic estimates (e.g. regression coefficient) AND variation (e.g. standard deviation) or associated estimates of uncertainty (e.g. confidence intervals)
☐	☒	For null hypothesis testing, the test statistic (e.g. F, t, r) with confidence intervals, effect sizes, degrees of freedom and P value noted<br>*Give P values as exact values whenever suitable.*
☒	☐	For Bayesian analysis, information on the choice of priors and Markov chain Monte Carlo settings
☒	☐	For hierarchical and complex designs, identification of the appropriate level for tests and full reporting of outcomes
☒	☐	Estimates of effect sizes (e.g. Cohen's d, Pearson's r), indicating how they were calculated
☐	☒	Clearly defined error bars<br>*State explicitly what error bars represent (e.g. SD, SE, CI)*

Our web collection on statistics for biologists may be useful.

Software and code

Policy information about availability of computer code

Data collection	Graph-Pad Software Inc, Version 5.01 was used for all data display.
Data analysis	Graph-Pad Software Inc, Version 5.01 was used for statistical analysis.

For manuscripts utilizing custom algorithms or software that are central to the research but not yet described in published literature, software must be made available to editors/reviewers upon request. We strongly encourage code deposition in a community repository (e.g. GitHub). See the Nature Research guidelines for submitting code & software for further information.

Data

Policy information about availability of data

All manuscripts must include a data availability statement. This statement should provide the following information, where applicable:

- Accession codes, unique identifiers, or web links for publicly available datasets
- A list of figures that have associated raw data
- A description of any restrictions on data availability

All data generated or analyzed during this study are included in this published article and its supplementary information files. The source data underlying Figs 1-10 and Supplementary Figs 1-15 are provided as two Source Data files.

Field-specific reporting

Please select the best fit for your research. If you are not sure, read the appropriate sections before making your selection.

☒ Life sciences ☐ Behavioural & social sciences ☐ Ecological, evolutionary & environmental sciences

For a reference copy of the document with all sections, see nature.com/authors/policies/ReportingSummary-flat.pdf

Life sciences study design

All studies must disclose on these points even when the disclosure is negative.

Sample size	No sample-size calculations was performed. Sample sizes are considered to be sufficient based on solid statistical results between groups.
Data exclusions	No data were excluded from the analyses.
Replication	Replicate experiments were successful.
Randomization	All mice sacrificed for experiment were not random. Mice analyzed were litter mates and sex-matched whenever possible.
Blinding	Investigators were not blinded to mouse genotypes and drug application during experiments. All image analyses, behavioral experiments, and other experiments were done blind to the experimental condition whenever possible. Data from no blinded experiments were not subjective but based on objectively acquired by related experimental software.

Reporting for specific materials, systems and methods

Materials & experimental systems

n/a	Involved in the study
☒	☐ Unique biological materials
☐	☒ Antibodies
☒	☐ Eukaryotic cell lines
☒	☐ Palaeontology
☐	☒ Animals and other organisms
☒	☐ Human research participants

Methods

n/a	Involved in the study
☒	☐ ChIP-seq
☒	☐ Flow cytometry
☒	☐ MRI-based neuroimaging

Antibodies

Antibodies used	The antibodies used for the experiments were provided in Antibodies and drugs section in Supplementary methods/pages 29-30 in Supplementary information .
Validation	Description of all antibodies used in this study can be found in Antibodies and drugs section in Supplementary methods/pages 29-30 in Supplementary information.

Animals and other organisms

Policy information about studies involving animals; ARRIVE guidelines recommended for reporting animal research

Laboratory animals	Description of research mice used for experiments can be found in the relevant figure legends, Animal section in Methods/page 24 in the main text, and Animal section in Supplementary Methods/pages 28-29 in Supplementary information.
Wild animals	No wild animal was used .
Field-collected samples	No field-collected sample was used.
